# Dietary Isoliquiritigenin at a Low Dose Ameliorates Insulin Resistance and NAFLD in Diet-Induced Obesity in C57BL/6J Mice

**DOI:** 10.3390/ijms19103281

**Published:** 2018-10-22

**Authors:** Youngmi Lee, Eun-Young Kwon, Myung-Sook Choi

**Affiliations:** 1 Department of Food Science and Nutrition, Kyungpook National University, 1370 San-Kyuk Dong Puk-Ku, Daegu 41566, Korea; 6k5rsg@hanmail.net (Y.L.); savage20@naver.com (E.-Y.K.); 2 Center for Food and Nutritional Genomics Research, Kyungpook National University, 1370 San-Kyuk Dong Puk-Ku, Daegu 41566, Korea

**Keywords:** isoliquiritigenin, insulin resistance, non-alcoholic fatty liver disease, energy expenditure, cytokine

## Abstract

Isoliquiritigenin (ILG) is a flavonoid constituent of *Glycyrrhizae* plants. The current study investigated the effects of ILG on diet-induced obesity and metabolic diseases. C57BL/6J mice were fed a normal diet (AIN-76 purified diet), high-fat diet (40 kcal% fat), and high-fat diet +0.02% (*w*/*w*) ILG for 16 weeks. Supplementation of ILG resulted in decreased body fat mass and plasma cholesterol level. ILG ameliorated hepatic steatosis by suppressing the expression of hepatic lipogenesis genes and hepatic triglyceride and fatty acid contents, while enhancing β-oxidation in the liver. ILG improved insulin resistance by lowering plasma glucose and insulin levels. This was also demonstrated by the intraperitoneal glucose tolerance test (IPGTT). Additionally, ILG upregulated the expression of insulin signaling-related genes in the liver and muscle. Interestingly, ILG elevated energy expenditure by increasing the expression of thermogenesis genes, which is linked to stimulated mitochondrial biogenesis and uncoupled cellular respiration in brown adipose tissue. ILG also suppressed proinflammatory cytokine levels in the plasma. These results suggest that ILG supplemented at 0.02% in the diet can ameliorate body fat mass, plasma cholesterol, non-alcoholic fatty liver disease, and insulin resistance; these effects were partly mediated by increasing energy expenditure in high-fat fed mice.

## 1. Introduction

High-fat diets and high-energy density foods such as fast foods, high-caloric beverages, and foods composed of refined grains, result in weight gain and obesity [1,2]. Obesity results from an imbalance between food intake and energy expenditure [3] and is defined medically as a state of increased adipose tissue [4]. White adipose tissue is the major site for storage of excess energy in the form of triglycerides (TG), releases free fatty acids (FFAs) for energy supply in fasting states, and secretes cytokines and other proteins involved in inflammatory responses [5]. Adipocyte hypertrophy and increased secretion of pro-inflammatory cytokines can contribute to a chronic inflammation. Also impaired TG deposition and increased lipolysis lead to the excess of circulating TG and FFAs that contribute to ectopic lipid accumulation including liver and skeletal muscle [6,7], thereby leading to hepatic steatosis and insulin resistance [8]. Consequently, obesity causes metabolic syndrome manifesting as dyslipidemia, insulin resistance, chronic low-grade inflammation, and non-alcoholic fatty liver disease (NAFLD) [3]. Hence, many studies have investigated the effects of a variety of natural phytochemicals on metabolic syndrome.

Isoliquilitigenin (ILG, 2′,4′,4-trihydroxychalcone) is a flavonoid with a chalcone structure [9]. ILG is abundant in the roots of a *Glycyrrhiza* species, which has been used as an oriental traditional medicine because of its various biological and pharmacological functions including anti-allergic, anti-tumor growth, anti-oxidation, and anti-angiogenesis effects [10,11]. Previous studies reported that ILG inhibits diet-induced adipose tissue inflammation by potently suppressing the nucleotide-binding domain, leucine-rich repeats containing family, pyrin domain-containing-3 inflammasome activation [11] and inhibiting high-fat diet-induced liver injury. ILG also suppresses hepatic steatosis by decreasing fat accumulation and repressing lipogenic genes in mice fed a high-fat diet [12]. ILG significantly attenuates protein expression of peroxisome proliferator-activated receptor γ, CCAAT/enhancer binding protein α, aP2, and glucose transporter type 4 in insulin-induced adipocyte differentiation. ILG inhibits lipid accumulation and inhibits the insulin-signaling pathway through protein-tyrosine phosphatase 1B activation, a negative regulator of the insulin signaling cascade in 3T3-L1 cells [13]. ILG also ameliorates plasma lipid levels, inhibits oxidative stress and inflammation, and attenuates atherosclerosis in apolipoprotein E-deficient mice [14]. ILG ameliorates caerulein-induced acute pancreatitis in ICR mice by decreasing oxidative stress injury of the pancreatic tissue, and increasing the proteins expression of Nrf2/HO-1 [15]. Moreover, ILG shows therapeutic effect on diabetic complication via increased SIRT1, thereby enhancing mitochondrial biogenesis, autophagy and anti-oxidant defense [16].

However, the effects of ILG on body fat mass and energy expenditure under obese conditions remains unclear, and to date, few studies have been conducted to investigate the anti-obesity effects of long-term supplementation with ILG. Therefore, we examined the role of long-term supplementation of ILG in alterations of body fat mass and energy expenditure, insulin resistance, NAFLD, and dyslipidemia in high-fat diet (HFD)-induced obesity using C57BL/6J mice as an animal model of obesity-related metabolic syndrome diseases.

## 2. Results

### 2.1. ILG Supplementation Lowered Liver and Adipose Tissues Weights and Plasma Total Cholesterol Levels

**Figure 1:**
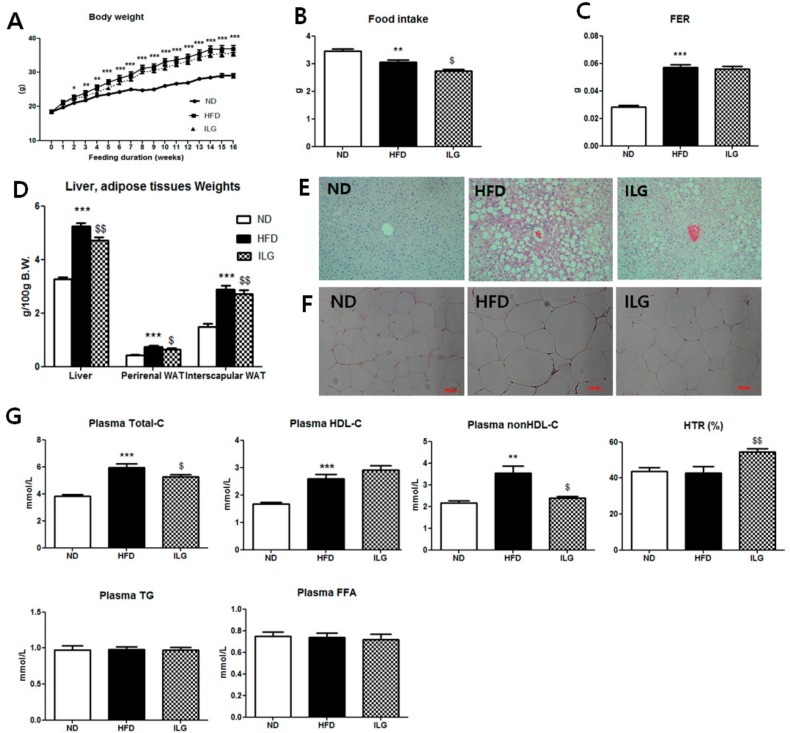
Effect of ILG supplementations on body weight (**A**), food intake; (**B**), food efficiency ratio (FER); (**C**), liver and adipose tissues weights; (**D**), hepatic morphology (magnification 200×); (**E**), WAT morphology (magnification 200×, scale bars indicate 50 μm); (**F**), and plasma lipids levels (**G**) in C57BL/6J mice fed a high-fat diet for 16 weeks. Mean ± SEM (ND (*n =* 13), HFD (*n =* 15), ILG (*n =* 13)). Significant differences between HFD versus ND are indicated; * *p* < 0.05, ** *p* < 0.01, *** *p* < 0.001. Significant differences between HFD versus ILG are indicated: ^$^ *p* < 0.05, ^$$^ *p* < 0.01. ND, normal diet (AIN-76); HFD, high-fat diet (40% kcal from fat; ILG, HFD + 0.02% (*w*/*w*) isoliquiritigenin; FER, food efficiency ratio, body weight gain/energy intakes per day; WAT, white adipose tissue; Total-C, total-cholesterol; HDL-C, high-density lipoproteins-cholesterol; nonHDL-C, total C − HDL-C; HTR, ratio of HDL-C to TC; TG, triglyceride; FFA, free fatty acid.

Supplementation of ILG did not affect body weight or the food efficiency ratio (FER), but decreased food intake compared to in the HFD group (Figure 1A–C). The weights of the liver and white adipose tissue (perirenal and interscapular WATs) were significantly higher in the HFD group than in the normal diet (ND) group, but ILG significantly lowered these weights compared to those in the HFD group. The average size in the epididymal white adipocyte of HFD group (9883.06 ± 481.06 μm^2^, *p* < 0.001) was larger than that of ND group (3239.07 ± 171.87 μm^2^). However, The epididymal white adipocyte size (6039.72 ± 391.93 μm^2^, *p* < 0.001) and hepatic lipid droplets in the ILG group were smaller than those in the HFD group (Figure 1D–F). The plasma total-cholesterol level was lower in the ILG group than in the HFD group, while non-HDL-cholesterol was significantly lowered, although ILG did not alter the plasma triglyceride (TG) and free fatty acid (FFA) levels. Concomitant with these results, the HDL-cholesterol/total-cholesterol ratio (HTR) was higher in the ILG group than in the HFD group (Figure 1G).

### 2.2. ILG Supplementation Lowered Hepatic Triglyceride and Fatty Acid, Hepatotoxicity, and Lipogenic-Related Enzyme Activity and Gene Expression

**Figure 2:**
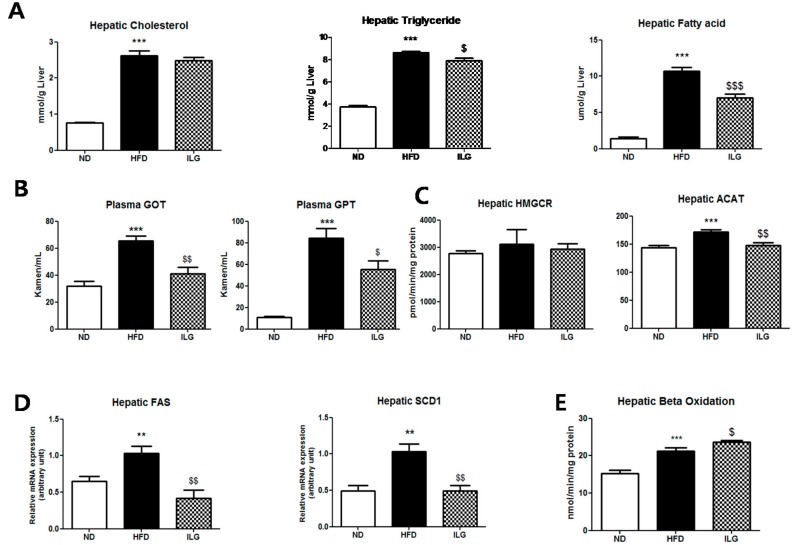
(**A**) Effect of ILG supplementation on hepatic lipid contents; (**B**) hepatic lipotoxicity markers; (**C**) hepatic lipid-regulating enzyme activities; (**D**) lipid-regulating gene expression; and (**E**) hepatic β-oxidation in C57BL/6J mice fed a high-fat diet for 16 weeks. Mean ± SEM (ND (*n* = 13), HFD (*n* = 15), ILG (*n* = 13)). Significant differences between HFD versus ND are indicated; ** *p* < 0.01, *** *p* < 0.001. Significant differences between HFD versus ILG are indicated: ^$^ *p* < 0.05, ^$$^ *p* < 0.01, ^$$$^ *p* < 0.001. ND, normal diet (AIN-76); HFD; high-fat diet (40 kcal% from fat); ILG, HFD + 0.02% (*w/w*) isoliquiritigenin; GOT, glutamic oxaloacetate transaminase; GPT, glutamic pyruvate transaminase; HMGCR, 3-hydroxy-3-methylglutaryl-coenzyme A (HMG-CoA) reductase; ACAT, acyl-CoA cholesterol acyltransferase; *FAS*, fatty acid synthase; *SCD1*, stearoyl-CoA desaturase 1.

ILG supplementation reduced hepatic triglyceride and fatty acid, and hepatotoxicity (plasma glutamic oxaloacetic transaminase (GOT) and glutamic pyruvic transaminase (GPT)) levels and suppressed hepatic acyl-CoA cholesterol acyltransferase (ACAT) activity and gene expression (fatty acid synthase (*FAS*) and stearoly-CoA desaturase 1 (*SCD1*)) levels compared to the HFD group (Figure 2A–D). However, ILG supplementation increased hepatic β-oxidation activity compared to in the HFD group (Figure 2E). There were no significant changes in hepatic cholesterol level (Figure 2A).

### 2.3. ILG Supplementation Ameliorated Insulin Resistance and Glucose Tolerance

**Figure 3:**
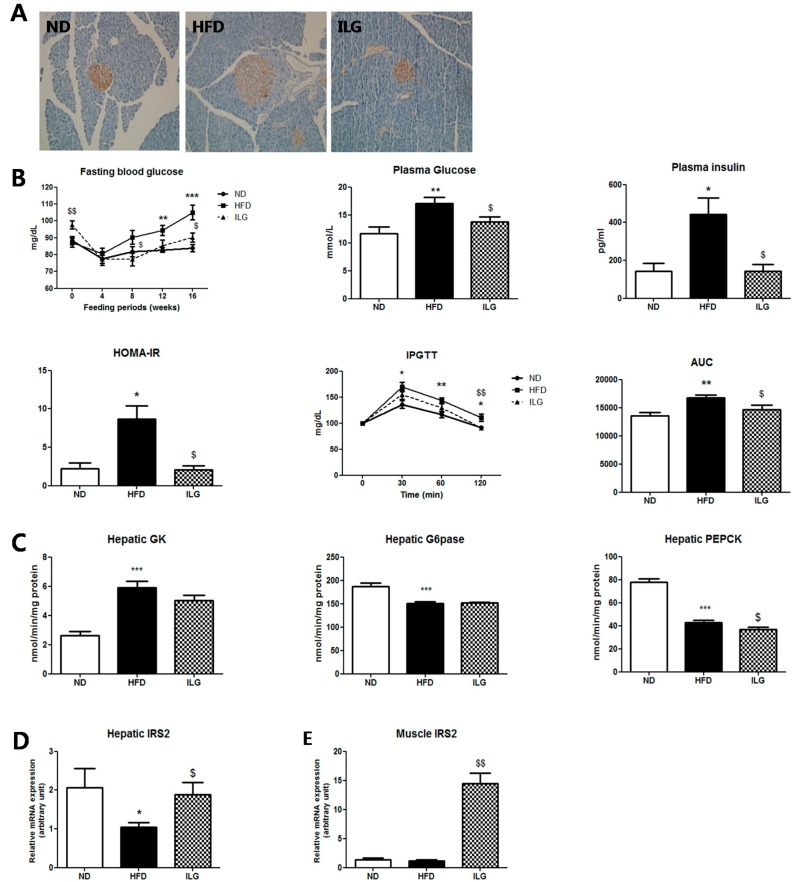
(**A**) Effect of ILG supplementation on pancreatic immunohistochemistry (magnification 200×); (**B**) fasting blood glucose, plasma glucose, plasma insulin, HOMA-IR, IPGTT, and AUC; (**C**) hepatic glucose-regulating enzyme activity; and (**D**,**E**) expression of IRS2 in the liver and muscle in C57BL/6J mice fed high-fat diet for 16 weeks. Mean ± SEM (ND (*n* = 13), HFD (*n* = 15), ILG (*n* = 13)). Significant differences between HFD versus ND are indicated; * *p* < 0.05, ** *p* < 0.01, *** *p* < 0.001. Significant differences between HFD versus ILG are indicated: ^$^ *p* < 0.05, ^$$^ *p* < 0.01. ND, normal diet (AIN-76); HFD; high-fat diet (40 kcal% from fat); ILG, HFD + 0.02% (*w/w*) isoliquiritigenin; HOMA-IR, homeostatic index assessment of insulin resistance; IPGTT, intraperitoneal glucose tolerance test; AUC, area under the concentration-time curve; GK, glucokinase; G6pase, glucose-6-phosphatase; PEPCK, phosphoenolpyruvate carboxykinase; *IRS*, insulin receptor substrate.

Immunohistochemistry staining showed that ILG supplementation significantly suppressed insulin secretion by the pancreatic islets compared to the HFD group (Figure 3A) and ameliorated insulin resistance with simultaneous decreases in fasting blood glucose, plasma glucose, and insulin, HOMA-IR, insulin resistance predictor, intraperitoneal glucose tolerance test (IPGTT), and area under the concentration-time curve (AUC) (Figure 3B). ILG reduced the activity of hepatic PEPCK, but not that of G6pase, which are gluconeogenic enzymes (Figure 3C); additionally, ILG significantly elevated the mRNA expression of insulin receptor substrate (*IRS*) *2* in both the liver and muscle (Figure 3D).

### 2.4. ILG Supplementation Increased Energy Expenditure and Expression of Insulin Signaling Genes in Muscle and Thermogenic Genes in Brown Adipose Tissue

**Figure 4:**
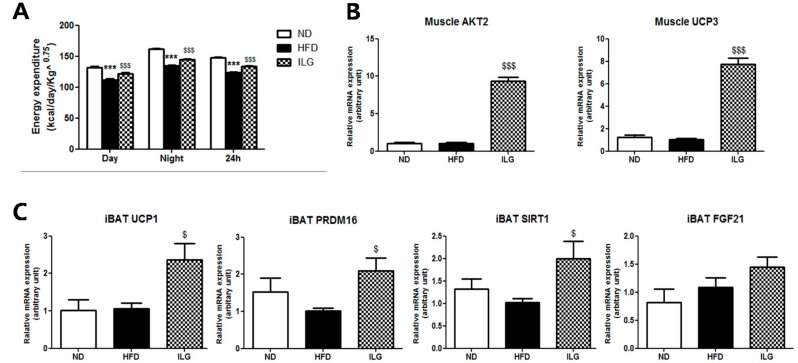
(**A**) Effect of ILG supplementation on energy expenditure; (**B**) muscle gene expression; and (**C**) interscapular BAT thermogenesis gene in C57BL/6J mice fed high-fat diet for 16 weeks. Mean ± SEM (ND (*n* = 13), HFD (*n* = 15), ILG (*n* = 13)). Significant differences between HFD versus ND are indicated; *** *p* < 0.001. Significant differences between HFD versus ILG are indicated: ^$^ *p* < 0.05, ^$$$^ *p* < 0.001. ND, normal diet (AIN-76); HFD, high-fat diet (40 kcal% from fat); ILG, HFD + 0.02% (*w/w*) isoliquiritigenin; iBAT, interscapular brown adipose tissue; *UCP 3*, uncoupling proteins 3; *PRDM16*, PR-domain containing 16; *SIRT1*, NAD-dependent deacetylase sirtuin-1; *FGF21*, fibroblast growth factor 21.

Energy expenditure was diminished in the HFD group compared to the ND group. However, ILG supplementation group markedly elevated energy expenditure (EE) during both the light phase and dark phase compared to in the HFD group (Figure 4A). Notably, ILG supplementation enhanced muscle *AKT2* and muscle uncoupling protein 3 (*UCP3*) gene expression (Figure 4B). Additionally, ILG significantly increased the mRNA expression of thermogenic genes, such as uncoupling protein 1 (*UCP1*), PR domain containing 16 (*PRDM16*), and NAD-dependent deacetylase sirtuin-1 (*SIRT1*) in the interscapular brown adipose tissue (iBAT) compared to in the HFD group, except for fibroblast growth factor 21 (*FGF21*) (Figure 4C).

### 2.5. ILG Supplementation Suppressed Inflammatory Adipokines

**Figure 5:**
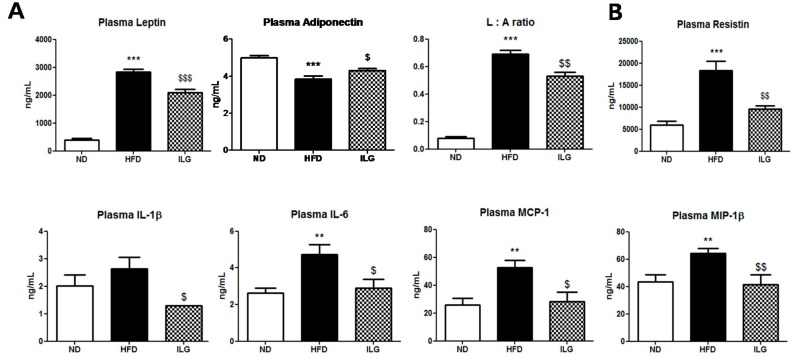
Effect of ILG supplementation on plasma adipokines (**A**) and plasma pro-inflammatory cytokines; (**B**) in C57BL/6J mice high-fat diet for 16 weeks. Mean ± SEM (ND (*n* = 13), HFD (*n* = 15), ILG (*n* = 13)). Significant differences between HFD versus ND are indicated; ** *p* < 0.01, *** *p* < 0.001. Significant differences between HFD versus ILG are indicated: ^$^ *p* < 0.05, ^$$^ *p* < 0.05, ^$$$^ *p* < 0.001. ND, normal diet (AIN-76); HFD; high-fat diet (40 kcal% from fat); ILG, HFD + 0.02% (*w/w*) isoliquiritigenin; L:A ratio, leptin: adiponectin ratio; IL, interleukin; MCP-1, monocyte chemoattractant protein-1; MIP-1β, macrophage inflammatory protein 1 beta.

Among plasma adipokines, HFD led to elevations of leptin, resistin, interleukin-6 (IL-6), monocyte chemoattractant protein-1 (MCP-1), and macrophage inflammatory protein 1 beta (MIP-1β) levels (Figure 5A,B). In contrast, plasma adiponectin level was lowered by the HFD (Figure 5A). The L:A ratio, a marker of metabolic disease and obesity, was decreased in the ILG group compared to in the HFD group (Figure 5A). Additionally, the levels of inflammatory cytokines, such as leptin, resistin, IL-1β, IL-6, MCP-1, and MIP-1β, were significantly attenuated by ILG supplementation (Figure 5B).

## 3. Discussion

In this study, we examined the effects of ILG on obesity-related metabolism in HFD-induced obese mice. Obesity leads to metabolic syndromes such as NAFLD, insulin resistance, hypertension, and cardiovascular disease. Our results showed that ILG did not alter body weights compared to in the HFD group (Figure 1A). However, in previous studies, when C57BL/6 mice consumed a HFD (60% fat (*w*/*w*) with ILG at an oral dose of 10 or 30 mg/kg/day, five times per week for the last five weeks of 11 weeks, body and liver weights were significantly reduced compared to in the HFD group without altering of food intake [12]. Additionally, C57BL/6 mice fed a HFD (60% fat) with ILG (0.5% *w*/*w*) for 20 weeks showed lower body weight gain compared to the HFD [11]. These differences may be partly related to differences in the dose and duration of ILG treatment. In the present study, food intake by the ILG group was lower than that by the HFD group and body weight was slightly reduced compared to in the HFD group (Figure 1A,B). Some medicinal plants have been shown to suppress appetite, prevent weight gain, and inhibit FAS and adipogenesis [17,18]. FAS inhibitors, such as cerulenin and C57, reduced food intake and body weight in mice [18]. Therefore, decreasing the weight of WAT and hepatic *FAS* mRNA by ILG supplementation seemed to be associated with a reduction in food intake.

ILG supplementation decreased hepatic FA and TG levels (Figure 2A). Furthermore, the ILG dose of 0.02% in the diet (*w*/*w*) was much lower than those used in previous studies. The decrease in liver weight following ILG supplementation may be related to the smaller lipid droplets observed (Figure 1D,E). In our study, ILG supplementation decreased plasma total cholesterol level, but not plasma TG and FFA levels (Figure 1G).

In general, the liver and the adipose tissue are the major tissues producing fatty acids [19]. FFAs play important roles in liver lipid metabolism. Plasma FFAs derived from dietary fat and FFAs by lipolysis of adipose tissue are transported to the liver via fatty acid-binding protein or Cd antigen 36/fatty acid translocase. These FFAs are processed via β-oxidation to generate energy or excessive FFAs accumulates in the form of triacylglycerol through esterification [19,20]. An imbalance between FFAs uptake and disposal eventually triggers hepatic steatosis, NAFLD, insulin resistance, and inflammation. C57BL/6 mice fed HFD developed NAFLD, insulin resistance, and fat accumulation in the pancreas [21]. Adipose tissue inflammation was observed after 24 weeks in HFD-fed C57BL/6J mice (45% kcal fat) [22]. In our study, hepatic fatty acid and triglyceride levels were decreased in the ILG group compared to in the HFD group through the suppression of *FAS* expression, although the plasma FFA level was unchanged (Figure 1G and Figure 2A,D). ILG supplementation resulted in increased hepatic β-oxidation activity and decreased lipogenic gene, *SCD1* (Figure 2D,E). These effects may have limited the availability of FFA in the liver, leading to decreased activity of the cholesterol-esterifying enzyme ACAT in ILG supplemented mice. These results indicate that ILG improved hepatic steatosis by lowering lipogenic enzyme activity and elevating β-oxidation activity. The decrease in hepatic fatty acid level appeared to be partly linked to the suppression of hepatic ACAT activity, as it is a substrate for esterification of hepatic free cholesterol. ILG did not alter hepatic cholesterol biosynthesis, as no changes were observed in HMG CoA-reductase activity, the rate-limiting enzyme of cholesterol biosynthesis. 

Lipogenesis partly depends on the insulin concentration and tissue insulin sensitivity [13]. We found that insulin resistance was associated with body fat mass in HFD-fed mice. HFD-fed mice showed substantial weight gain and elevated plasma blood glucose and insulin levels. Pancreatic islets of the HFD-fed mice were also enlarged. Compensatory hyperplasia of pancreatic islets plays a critical role in delaying hyperglycemia [23]. Interestingly, ILG normalized pancreatic β-cells and improved fasting blood glucose, plasma glucose and insulin levels, and decreased the HOMA-IR level (Figure 3A,B). Under conditions of insulin resistance, gluconeogenesis can be increased via G6Pase or PEPCK activity [24]. ILG partly suppressed gluconeogenesis and increased the expression of hepatic *IRS2* mRNA (Figure 3C,D), although hepatic AMP-activated protein kinase was not measured. 

Skeletal muscle plays an important role in glucose uptake as one of major target organs of insulin actions [25,26]. ILG appeared to improve insulin resistance by increasing *IRS2* and *AKT2* mRNA levels in the skeletal muscle (Figure 4B). This is supported by previous results obtained in *ir/irs-2^+/−^* transgenic mice, which developed insulin resistance in the liver and skeletal muscle and modest β-cell hyperplasia [27]. Insulin-mediated *AKT* was decreased in HFD-induced obesity [25], and *AKT2^−/−^* mice developed peripheral insulin resistance and showed hepatic glucose production [26]. According to our findings, ILG regulates energy expenditure and thermogenesis. *UCP3* is a highly skeletal muscle-specific protein and differentially regulated from BAT *UCP1* as reported in studies of cold adaptation [28]. When transgenic mice overexpressing *UCP3* in the skeletal muscle were fed HFD, a 30% increase in total energy expenditure and 60% increase in insulin-stimulated AKT2 activity was observed compared to HFD-fed wild-type mice [29]. These mice exhibited increased insulin-stimulated glucose uptake. Our results also support that the role of *UCP3* in skeletal muscle is to improve insulin resistance and increase energy expenditure (Figure 4A,B). ILG also significantly upregulated the mRNA expression of thermogenic genes, such as *UCP1*, *PRDM16*, and *SIRT1* in iBAT (Figure 4C). BAT and skeletal muscle are important contributors to peripheral energy expenditure and counteract obesity [30]. Proliferator-activated receptor gamma -γ coactivator-1α (PGC-1α), a key regulator of energy metabolism [31], activates UCP1 which dissipate the proton gradient across the inner mitochondrial membrane for generating heat at the expense of ATP [32]. *PRDM16* is expressed at higher levels in brown adipocytes compared to in white adipocytes, stimulates mitochondrial biogenesis and uncoupled cellular respiration, and increases the expression of *PGC-1α* and *UCP1* [33]. SIRT1 is an NAD^+^-dependent protein deacetylase [34]. SIRT1-activating compounds such as resveratrol and SIRT1720 increase mitochondrial biogenesis in the liver and skeletal muscle and improve insulin sensitivity in obese mice [35]. Other compounds that exhibit the same effect share structural similarity to resveratrol. These include the chalcones butein and isoliquiritigenin [36]. *SIRT1* overexpression results in increased energy expenditure [37]. Although the expression of *FGF21* was not significantly different in our experiment, systemic administration of FGF21 in mice resulted in increased energy expenditure in a previous study [38]. Furthermore, BAT activation improved glucose tolerance and insulin sensitivity via UCP1, PRDM16, and FGF21 [38]. Consistent with these observations, ILG increased insulin sensitivity and energy expenditure, decreased adiposity, and upregulated the thermogenesis-related genes (*UCP1*, *PRDM16*, and *SIRT1*) in iBAT.

Our results also indicate that ILG supplementation significantly attenuated the levels of plasma inflammatory markers and insulin resistance. In type 2 diabetes mellitus and metabolic syndrome, pro-inflammatory cytokines linked to insulin resistance and low-grade chronic inflammation are elevated, while the anti-inflammatory molecule adiponectin is decreased [33]. Leptin upregulates IL-6 and tumor necrosis factor-α [39,40], and resistin and IL-6 partly regulate blood glucose [41]. In contrast, adiponectin ameliorates insulin resistance [42] and stimulates β-oxidation of fatty acids in myocytes [43]. In general, the plasma leptin: adiponectin ratio is a useful measure of insulin resistance and predictor the presence of metabolic syndrome [44]. These studies indicate that ILG supplementation potently ameliorates HFD-induced insulin resistance which is partly mediated by alterations in plasma leptin, adiponectin, resistin, and the L:A ratio. IL-1β is produced mostly by adipose tissue macrophages and its release is increased in obesity [45]. IL-1β is involved in mediating macrophage adipocyte crosstalk during insulin signaling in human adipocytes, and macrophage-derived factors markedly increase the production of pro-inflammatory cytokines/chemokines (i.e., IL-6, MCP-1, CCL-5, IL-8) by adipocytes as well as preadipocytes [46]. In patients with NAFLDs, serum MCP-1 gradually increases with development of nonalcoholic steatohepatitis and contributes to insulin resistance in the skeletal muscle [47]. Consistent with MCP-1, MIP-1β is an inflammatory chemokine that may be up-regulated in patients with type 2 diabetes and cardiovascular disease [48].

## 4. Materials and Methods

### 4.1. Animals and Diets

Four-week-old male C57BL/6J mice (*n* = 41) were obtained from the Jackson Laboratory (Bar Harbor, ME, USA). All mice were housed individually at a constant temperature of 24 °C, humidity of 40%, and 12-h light/dark cycle. After a one-week acclimation period, the mice were randomly assigned to three groups and fed experimental diets for 16 weeks: normal diet (AIN-76A purified diet, *n =* 13), high-fat-diet (20% fat based on AIN-76A purified diet plus 1% cholesterol, *w*/*w*, *n =* 15) and high-fat-diet +0.02% (*w*/*w*) ILG (*n* = 13). ILG was isolated from the roots of the *Glycyrrhiza Uralensis Fisch* (Chengdu Biopurify Phytochemicals, Ltd., Chengdu, China). The HFD contained 40.4 kcal% fat, 17.4 kcal% protein, 41.2 kcal% carbohydrate, and 1 kcal% vitamin mix. For dietary fat sources, 85% (*w*/*w*) of total fat was lard and 15% (*w*/*w*) was corn oil. The mice had free access to food and distilled water. Food consumption and body weight were measured daily and weekly, respectively, and blood glucose levels were measured every four weeks. At the end of the experimental period, all mice were anesthetized with isoflurane (5 mg/kg body weight, Baxter, San Juan, Puerto Rico) after a 12-h fast. Blood was collected from the inferior vena cava for the determination of plasma lipid, glucose, insulin, and cytokine levels. After blood collection, the pancreas, liver, adipose tissue, and muscle were removed, rinsed with physiological saline, weighed, immediately frozen in liquid nitrogen, and stored at −70 °C until analysis. The animal study protocols were approved by the Ethics Committee of Kyungpook National University (approval no. KNU 2016-37, 7 March 2016).

### 4.2. Energy Expenditure

Energy expenditure (EE) was measured at 14 weeks using an indirect calorimeter (Oxylet; Panlab, Cornella, Spain). The mice were placed into individual metabolic chambers at 22 ± 2 °C, with free access to food and water. The O_2_ and CO_2_ analyzers were calibrated with high-purity gas. Oxygen consumption (VO_2_) and carbon dioxide production (VCO_2_) were recorded at 3-min intervals using a computer-assisted data acquisition program (Chart 5.2; AD Instrument, Sydney, Australia) over a 24-h period, and the data were averaged for each mouse. EE was calculated according to the following formula: EE (kcal/day/body weight^0.75^) = VO_2_ × 1.44 × [3.815 + (1.232 × VO_2_/VCO_2_)].

### 4.3. Blood Analysis

Plasma triglyceride, total cholesterol, high-density lipoproteins (HDL)-cholesterol, glucose, glutamic oxaloacetic transaminase, and glutamic pyruvic transaminase levels were determined using commercial kits (Asan Pharm, Seoul, Korea) and the concentration of plasma FFA was measured using a commercial kit from Wako (Osaka, Japan). All assays were performed according to the manufacturer’s instructions. Values of non-HDL-cholesterol and HDL-cholesterol to total cholesterol ratio (HTR) were calculated as follow: non-HDL-cholesterol = (total-cholesterol) − (HDL-cholesterol); HTR (%) = (HDL-cholesterol/total-cholesterol) × 100%. Plasma hormone (insulin) and adipokines (leptin and resistin) and cytokines (interleukin-1 beta (IL-1β), IL-6, monocyte chemoattractant protein-1 (MCP-1), and macrophage inflammatory protein 1 beta (MIP-1β) were measured with a multiplex detection kit (MILLIPLEX^®^_MAP_ Mouse metabolic magnetic bead panel and MILLIPLEX^®^_MAP_ Mouse cytokine/chemokine magnetic bead panel (Merck, Darmstadt, Germany). All samples analyzed with a Luminex 200 and ×PONENT software. Median Fluorescent Intensity (MFI) Data was analyzed using a five-parameter logistic for calculating analyte concentrations. Adiponectin was measured with a Quantikine MILLIPLEX^®^ ELISA kit (R and D Systems, Minneapolis, MN, USA).

### 4.4. Fasting Blood Glucose, Intraperitoneal Glucose Tolerance Test (IPGTT), and Homeostatic Index of Insulin Resistance (HOMA-IR)

The fasting blood glucose concentration was measured every 4 weeks by the glucose oxidase method using a glucometer (glucDr super sensor, Allmedicus, Anyang, Korea) in whole blood obtained from the tail vein after food withholding for 12 h. Fasting plasma glucose levels were determined using a commercial kit (Asan Pharm, Seoul, Korea). The homeostatic index of insulin resistance (HOMA-IR) was calculated according to the homeostasis assessment model as follows: HOMA-IR = [(fasting plasma glucose (mg/dL) × 0.05551) × fasting plasma insulin (IU/mL)]/22.51. An IPGTT was performed at 12th week. The mice were fasted for 12 h, and then injected intraperitoneally with glucose (0.5 g/kg body weight). The body glucose level was determined from the tail vein at 0, 30, 60, and 120 min using a glucometer after glucose injection. AUC was calculated using NCSS statistical software.

### 4.5. Lipid Analysis in the Liver

Hepatic lipids were extracted [49], and dried lipid residues were dissolved in 1 mL of ethanol for the cholesterol, triglyceride, and fatty acid (FA) assays. Triton X-100 and a sodium cholate solution in distilled water were added to 200 µL of a dissolved lipid solution for emulsification. Hepatic lipids were analyzed using the same enzymatic kits used for plasma analyses.

### 4.6. Morphological Analysis of Liver, Epididymal WAT, and Pancreas

The liver, epididymal white adipose tissue (WAT), and pancreas were excised from each mouse. Samples were fixed in 10% (*v*/*v*) paraformaldehyde/phosphate-buffered saline. The liver and epididymal WAT were embedded in paraffin for staining with hematoxylin and eosin. For immunohistochemistry, pancreas was stained for determining B-cell with avidin-biotin complex. After contrast dyeing with Mayer’s hematoxylin, the cells were dehydrated, clarified, and sealed. The stained slices were examined under an optical microscope (Nikon ECLIPSE N*i*, Tokyo, Japan) at 200× magnification. To measure adipocyte size, photomicrograph was taken on a Zeiss Axioskop 2 (Zeiss, Oberkochen, Germany) at 200× magnification using Axiovision SE64 software.

### 4.7. Measurement of Enzyme Activity

Hepatic mitochondrial, cytosolic, and microsomal fractions were prepared as described by Hulcher and Oleson [50] with slight modifications. Protein concentrations were determined using the Braford method with bovine serum albumin as the standard [51]. Mitochondrial fatty acid β-oxidation was measured by monitoring the reduction of NAD^+^ to NADH at 340 nm as described by Lazarow [52]. Cytosolic glucokinase activity was measured using a spectrophotometric method as described by Davidson and Arion [53]. Microsomal glucose-6-phosphatase (G6Pase) activity was measured with a spectrophotometric assay according to the method of Alegre et al. [54]. Cytosolic phosphoenolpyruvate carboxykinase (PEPCK) activity was monitored in the direction of oxaloacetate synthesis using the spectrophotometric assay described by Bentle and Lardy [55]. Microsomal HMG-CoA reductase (HMGCR) and acyl-CoA cholesterol acyltransferase (ACAT) activities were measured with [^14^C] HMG-CoA [56] and [^14^C]oleoyl-CoA [57] as substrates, respectively.

### 4.8. RNA Isolation and Real-Time Quantitative Reverse Transcription-Polymerase Chain Reaction

Total RNA was extracted from the liver, muscle, and interscapular brown adipose tissue (BAT) using TRIsol reagent (Invitrogen Life Technologies, Carlsbad, CA, USA) according to the manufacturer’s instructions. DNase digestion was used to remove any DNA contamination and RNA was re-precipitated in ethanol to ensure no phenol contamination. For quality control, RNA purity and integrity were evaluated using the Agilent 2100 Bioanalyzer (Agilent Technologies, Santa Clara, CA, USA). Equal amount of RNA from each of the study groups were pooled to normalize individual differences. The total RNA was converted to cDNA using the QuantiTect Reverse Transcription Kit (Qiagen Hilden, Germany). mRNA expression was quantified by a quantitative real-time polymerase chain reaction (PCR) using the QuantiTect SYBR Green PCR kit (Qiagen) and the CFX96 real-time system (Bio-Rad, Hercules, CA, USA). Each cDNA sample was amplified using primers for the glyceraldehyde-3-phosphate dehydrogenase (*GAPDH*) gene labeled with SYBR green dye. Amplification was performed as follows: 10 min at 90 °C, and 60 s at 60 °C for a total of 40 cycles. The cycle threshold (*C*_t_) was obtained as the cycle which a statistically significant. Using the 2^ΔΔ*C*t^, the fold-changes were calculated.

### 4.9. Primers

The primer pairs were as follows: *FAS* (Forward: 5′-GCT GCG GAA ACT TCA GGA AAT-3′, Reverse: 5’-AGA GAC GTG TCA CTC CTG GAC TT-3’), *SCD1* (Forward: 5′-CCC CTG CGG ATC TTC CTT AT-3′, Reverse: 5′-AGG GTC GGC GTG TGT TTC T-3′), *IRS2* (Forward: 5′-CAC AAG TAC CTG ATC GCC CTC TAC-3′, Reverse: 5′-CTC CTG CTC CTG CTC GTT CTC-3′), *AKT2* (ACG TGG TGA ATA CAT CAA GAC C-3’, Reverse: 5′-GCT ACA GAG AAA TTG TTC AGG GG-3′), *UCP3* (Forward: 5′-GGA TTT GTG CCC TCC TTT CTG-3′, Reverse: 5′-AGA TTC CCG CAG TAC CTG GAC-3′), *UCP1* (Forward: 5′- AGA TCT TCT CAG CCG GAG TTT-3′, Reverse: 5′-CTG TAC AGT TTC GGC AAT CCT-3′), *PRDM16* (Forward: 5′-AGG GCA AGA ACC ATT ACA CG-3′, Reverse: 5′-GGA GGG TTT TGT CTT GTC CA-3′), *SIRT1* (Forward: 5′-TGT GAA GTT ACT GCA GGA GTG TAA A-3′, Reverse: 5′-GCA TAG ATA CCG TCT CTT GAT CTG AA-3′), *FGF21* (Forward: 5′-ATG GAA TGG ATG AGA TCT AGA GTT GG-3′, Reverse: 5′-TCT TGG TGG TCA TCT GTG TAG AGG-3′).

### 4.10. Statistical Analysis

The parameter values were expressed as the mean ± standard error mean (SEM). Significant differences between the ND and HFD groups and between the HFD and ILG groups were assessed by Student’s *t* tests. Statistical analysis was carried out using SPSS version 22 software (SPSS, Inc., Chicago, IL, USA). The results were considered statistically significant at *p* < 0.05.

## 5. Conclusions

**Figure 6:**
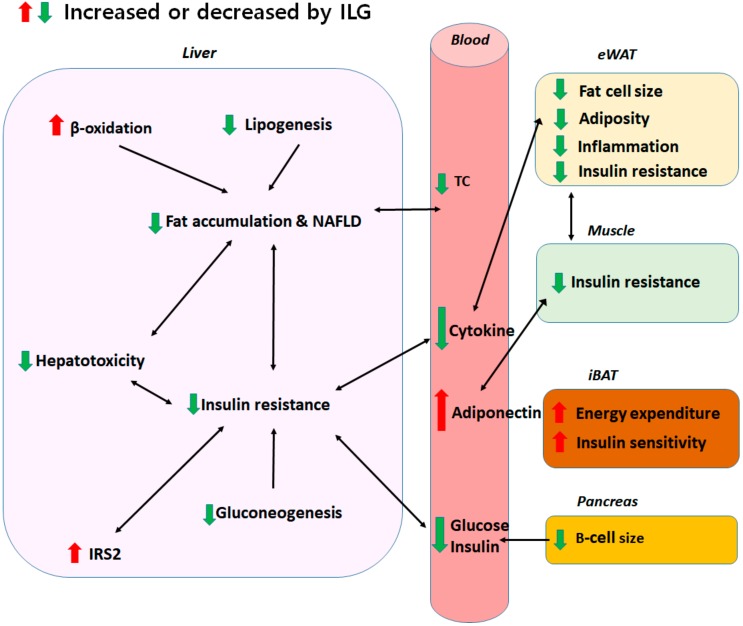
Schematic representation of the effects of ILG on diet-induced obesity and metabolic diseases. ILG suppressed accumulation of hepatic TG and FA and ameliorates NAFLD by decreasing hepatic lipogenesis, while increasing β-oxidation, thereby preventing hepatotoxicity. Additionally, ILG improved insulin resistance as evidenced by reduced plasma glucose and insulin levels and normalized the pancreatic B-cell size via reducing hepatic gluconeogenesis and plasma pro-inflammatory cytokine levels, as well as by enhancing hepatic and muscle IRS2 mRNA and muscle AKT mRNA expressions. Reduced plasma inflammatory cytokine levels, decreased fat cell size in eWAT, and elevated adiponectin attenuated adiposity and inflammation. ILG increased energy expenditure and thermogenesis by increasing UCP1, PRDM16, and SIRT1 mRNA expression in iBAT, which also contributes to the prevention of insulin resistance.

Our results provide insight into the role of ILG, which was found to modulate obesity-associated metabolic disorders such as hepatic steatosis and insulin resistance. ILG lowered the liver and, in part, adipose tissue weights and plasma total cholesterol level. It suppressed accumulation of hepatic triglycerides and fatty acids by regulating lipogenesis-related enzyme activities and the expression of some genes. These results suggest that ILG ameliorates adiposity and NAFLD. Furthermore, ILG improves insulin resistance by suppressing gluconeogenesis and increasing mRNA *IRS2* expression and suppresses inflammatory cytokine levels. ILG also increases energy expenditure by increasing expression of muscular and interscapular brown adipose thermogenic genes. Figure 6 summarizes the effects of ILG on diet-induced obesity and metabolic diseases. Taken together, these findings indicate that IL is a useful natural candidate for controlling obesity-related metabolic disorders such as adiposity, NAFLD, insulin resistance, and inflammation.

## Abbreviations

NAFLD	Non-alcohol fatty liver disease
IPGTT	Insulin and intraperitoneal glucose tolerance test
FER	Food efficiency ratio
HDL	High-density lipoproteins
TG	Triglyceride
FFA	Free fatty acid
HTR	HDL-cholesterol/total-cholesterol ratio
EE	Energy expenditure
WAT	White adipose tissue
GOT	Glutamic oxaloacetic transaminase
GPT	Glutamic pyruvic transaminase
ACAT	Acyl-CoA cholesterol acyltransferase
FAS	Fatty acid synthase
SCD1	Stearoyl-CoA desaturase 1
HMGCR	3-Hydroxy-3-methylglutaryl-coenzyme A (HMG-CoA) reductase
PEPCK	Phosphoenolpyruvate carboxykinase
GK	Glucokinase
G6pase	Glucose-6-phosphatase
HOMA-IR	Homeostatic index Assessment for Insulin Resistance
AUC	Area under the concentration-time curve
IRS2	Insulin receptor substrate 2
UCP3	Uncoupling protein 3
UCP1	Uncoupling protein 1
iBAT	Interscapular brown adipose tissue
PRDM16	PR domain containing 16
SIRT1	NAD-dependent deacetylase sirtuin-1
FGF21	Fibroblast growth factor 21
L:A ratio	Leptin:adiponectin ratio
IL-1β	Interleukin 1 beta
IL-6	Interleukin 6
MCP-1	Monocyte chemoattractant protein-1
MIP-1β	Macrophage inflammatory protein 1 beta
